# Results on patient-reported outcomes are underreported in summaries of product characteristics for new drugs

**DOI:** 10.1186/s41687-021-00402-1

**Published:** 2021-12-07

**Authors:** Susanne Haag, Lisa Junge, Fabian Lotz, Natalie McGauran, Marios Paulides, Regine Potthast, Thomas Kaiser

**Affiliations:** 1 Institute for Quality and Efficiency in Health Care, Cologne, Germany; 2 Drug Commission of the German Medical Association, Berlin, Germany

## Abstract

**Background:**

Summaries of product characteristics (SmPCs) are regulatory documents published upon drug approval. They should report all relevant study data and advise how to use drugs safely and effectively. Patient-reported outcomes (PROs) are increasingly used in clinical trials to incorporate the patient perspective—SmPCs should thus adequately report PROs. In Germany, new drugs undergo mandatory early benefit assessment. Pharmaceutical companies submit dossiers containing all evidence; the subsequent dossier assessments focus on patient-relevant outcomes and comprehensively report PROs.

**Objective:**

The primary aim was to investigate to what extent PROs recorded as outcomes in clinical trials of new drugs are reported in SmPCs.

**Methods:**

We analysed dossier assessments with randomized controlled trials (RCTs) of new drugs entering the market between 01/2014 and 07/2018 and the corresponding SmPCs, and compared PRO reporting in both document types. For this purpose, we evaluated dossier assessment characteristics (e.g. drug name, indication, disease category) and study characteristics (e.g. evaluable PROs available?). PROs were divided into symptoms and health-related quality of life (HRQoL). SmPCs were screened to identify RCTs. We conducted 3 main evaluation steps: (1) Did the RCT included in the dossier assessment contain evaluable PROs? (2) If yes, was the RCT included in the SmPC? (3) If yes, were the PROs reported in the SmPC? Results are presented descriptively.

**Results:**

88 dossier assessments including 143 RCTs on 72 drugs were considered: 109 (76.2%) RCTs included evaluable PROs, of which 89 were included in SmPCs. 38 RCTs (42.7%) investigated oncologics, 18 (20.2%) anti-infectives, and 33 (37.1%) other drugs. The RCTs considered symptoms more often than HRQoL (82 vs. 66 RCTs). In SmPCs, PROs were reported for 41 RCTs (46.1%), with a slightly higher reporting rate for RCTs considering HRQoL (43.9%) than for RCTs considering symptoms (41.5%). In oncologic indications, PROs were reported for 36.7% of RCTs considering HRQoL and 33.3% of RCTs considering symptoms. In infectious diseases, the rates were 21.4% (symptoms) and 0% (HRQoL), and for other diseases about 60% (symptoms) to 70% (HRQoL).

**Conclusion:**

Even though a large amount of PRO data on new drugs is available from clinical trials included in SmPCs, the corresponding results are underreported.

**Supplementary Information:**

The online version contains supplementary material available at 10.1186/s41687-021-00402-1.

## Background

Clinical trials used for regulatory approval provide essential information on new drugs. Traditionally, they primarily focus on objective clinical outcomes and laboratory parameters. However, the importance of considering the patient perspective to fully capture the benefits and harms of new drugs as a basis for regulatory, health policy, clinical and patient-centred decision-making is now widely accepted. Patient-reported outcomes (PROs) are therefore also increasingly being used in clinical trials to measure effectiveness [1–6].

PROs cover both single and multi-dimension measures of outcomes such as symptoms and health-related quality of life (HRQoL). They “provide a unique means of capturing the personal and social context of the disease and treatment experience, as OS (overall survival), PFS (progression-free survival), biomarker measures or adverse events may not necessarily capture the full impact of a treatment on how a patient feels or functions” [2].

In Europe, a drug’s marketing authorization includes the creation of a legal document called a summary of product characteristics (SmPC), which is published at the time of market authorization or extension of therapeutic indication. The SmPC provides healthcare professionals with information on the drug’s properties, approved conditions of use, safe and effective use, and main results of clinical trials supporting market authorization. It is updated regularly to include new data (e.g. on safety) [7–9].

In the United States, the equivalent of the SmPC is the prescribing information (also called product information or labelling or package insert) [10].

In Germany, an early benefit assessment according to the Act on the Reform of the Market for Medicinal Products (AMNOG) is conducted for every new drug after market entry and for previously approved patented drugs with new therapeutic indications [11]. The Federal Joint Committee (G-BA), the main decision-making body in the healthcare system, generally commissions the Institute for Quality and Efficiency in Health Care (IQWiG) for this purpose. In the early benefit assessment, symptoms and HRQoL are always considered as essential patient-relevant outcomes besides mortality and adverse effects. The target population investigated is not necessarily identical to the one defined in an SmPC, as specific aspects of the German healthcare setting may be considered (e.g. a subpopulation receiving the standard care defined by the G-BA). All data on a new drug, including the clinical study reports, must be submitted in a dossier by the manufacturer and all relevant study results are published online in a so-called dossier assessment, including those on PROs [11, 12].

In line with regulatory requirements [2, 3], only PROs recorded with valid and suitable tools are considered in dossier assessments in Germany; we would thus expect these PROs, at least the ones included in the main trials supporting market authorization, to be reported in SmPCs.

### Objective

Our primary aim was to investigate to what extent PROs recorded as outcomes in clinical trials used for regulatory approval are reported in SmPCs of new drugs. We therefore compared the reporting of PROs in dossier assessments with the reporting of PROs in the corresponding SmPCs. We also investigated whether the reporting of PROs in SmPCs depended on the direction of the treatment effect.

## Methods

### Data sources

Eligible dossier assessments were identified via IQWiG’s internal (non-public) AMNOG database. All assessments were considered that included randomized controlled trials (RCTs) and assessed drugs entering the market or receiving an extension of therapeutic indication between 01.01.2014 and 01.07.2018. Orphan drugs were excluded: orphan drug status is automatically associated with a (fictitious) added benefit; only the extent of added benefit is assessed, primarily by the G-BA. The dossier assessments were downloaded from the G-BA website [13].

The corresponding SmPCs (including any updates) were identified via the EU Register of medicinal products [14].

### Data extraction and evaluation

We extracted and evaluated the following information from the dossier assessments:

- *Characteristics of the dossier assessment* project number, drug name, therapeutic indication (i.e. target disease and target population), and disease category.
- Study characteristics:
  - name of RCT
  - features of RCT:
    - whether it investigated the target population specified in the SmPC or a different population (specified in the dossier assessment), including the reason for this deviation;
    - whether it included evaluable PROs, i.e. sufficient data recorded with valid and suitable tools.
- From each RCT included in the dossier assessment, the PROs were evaluated separately for the outcome categories of morbidity (symptoms) and HRQoL, according to the classification in the dossier assessments.

The corresponding SmPCs were screened to identify the RCTs included in the dossier assessment. The PROs included in these RCTs and reported in the SmPCs were then evaluated as described above.

Data were extracted and evaluated by one author (LJ) according to the table in Additional file 1 and then checked by another (SH). Any discrepancies were resolved by consensus; overall, discrepancies in data evaluation between the two authors were low (< 10%).

### Information synthesis and analysis

We first compiled the information extracted, followed by a 5-step procedure:

1. Did the RCT included in the dossier assessment contain evaluable PRO data?
  yes/no; per outcome category
2. If yes, was the RCT also included in the SmPC?
  yes/no
3. If yes, were the PROs that were reported in the dossier assessment also reported in the SmPC?
  yes/no; per outcome category and disease category

The following steps only considered those RCTs where the whole study population or a subpopulation specified in the therapeutic indication of the SmPC was included in the dossier assessment investigating this target population (e.g., a subpopulation with high disease severity), as in these cases, the PROs reported in the SmPC were expected to be consistent with the PROs reported in the dossier assessment.

4. To what extent were the PROs reported in the SmPC?
  completely/partly/not reported; per outcome category
5. To what extent were the PROs reported in the SmPC according to the direction of the treatment effect?
  completely/partly/not reported; per outcome category and effect category (positive vs. negative/no effect—the latter was combined post hoc due to the small number of RCTs showing a negative effect, see Additional file 2).

All results are presented descriptively without further statistical testing.

## Results

### Document and study characteristics

**Fig. 1:**
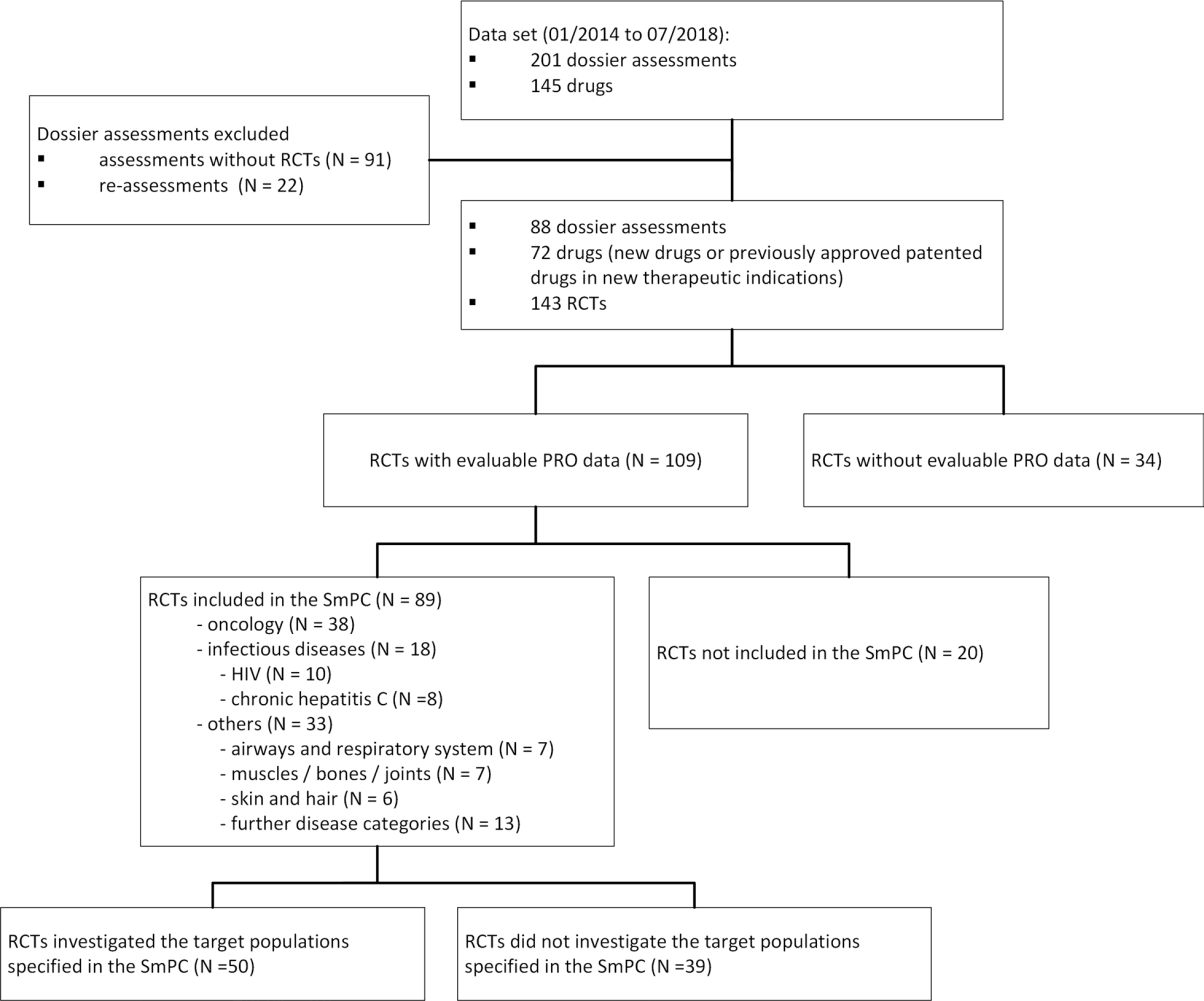
Flow chart of the data set included. *HIV* human immunodeficiency virus, *N* number of RCTs, *PRO* patient-reported outcome, *RCT* randomized controlled trial, *SmPC* summary of product characteristics

88 dossier assessments including 143 RCTs on 72 drugs were considered (Fig. 1). 109 (76.2%) of these RCTs included evaluable PRO data, of which 89 were included as main trials in the SmPCs: 38 RCTs (42.7%) investigated oncologics, 18 (20.2%) anti-infectives (all for HIV or chronic hepatitis C), and 33 (37.1%) other drugs. For 50 of the 89 RCTs, the population investigated in the dossier assessments corresponded to the target population specified in the SmPC.

### Reporting rates for PROs in SmPCs

#### Overall results

**Fig. 2:**
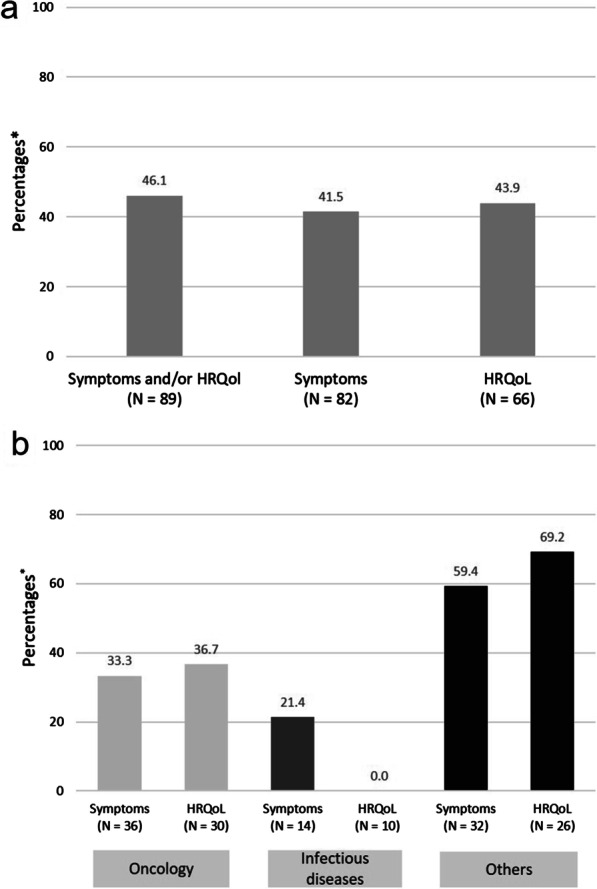
Reporting rate of PROs in SmPCs: **a** by outcome category, **b** by outcome/disease category. *Proportion of RCTs with evaluable PRO data reported in the SmPCs. *HRQoL* health-related quality of life, *N* number of RCTs with evaluable PRO data included in dossier assessments, *PRO* patient-reported outcome, *SmPC* summary of product characteristics

PRO data were reported for 41 out of 89 RCTs (46.1%) considering evaluable PROs and included in the SmPCs (Fig. 2a). In the 89 RCTs, PROs on symptoms were investigated more often than those on HRQoL, but the reporting rate for PROs from RCTs considering HRQoL was slightly higher.

Figure 2b shows reporting according to disease category: The reporting rate was lowest for PROs in infectious diseases (PRO data were reported for 21.4% of RCTs considering symptoms and 0% of RCTs considering HRQoL). In oncologic indications, PRO data were reported for 33.3% of RCTs considering symptoms and 36.7% of RCTs considering HRQoL. In other disease categories, PRO data were reported for about 60% (symptoms) to 70% (HRQoL) of the RCTs.

#### RCTs investigating the target population specified in SmPCs

The reporting rate was even lower for the PROs from the 50 RCTs covering the target population specified in the SmPCs. PRO data were fully or partly reported only for 32.6% (symptoms) and 25.8% (HRQoL) of these RCTs (Additional file 3).

#### Selective outcome reporting

The data investigating whether the extent of reporting in SmPCs depended on the direction of the treatment effect on the PROs are shown in Additional file 3. For RCTs considering symptoms, PRO data were reported for 50% of the RCTs showing positive effects, whereas this was the case only for 18% of RCTs showing negative or no effects. However, for RCTs considering HRQoL, there were only small differences in the corresponding reporting rates.

## Discussion

Despite endeavours to incorporate the patient perspective in drug development and regulatory decision-making, a substantial amount of information on PROs considered in RCTs on new drugs is not reported in the corresponding SmPCs. In the two disease categories with sufficient data for separate analysis, oncology and infectious diseases, reporting of PROs was poor for both categories and particularly poor for the latter. An explanation could be that the assessment of treatment benefit in infectious diseases is traditionally based on biomarkers [15].

No consistent difference was shown between reporting rates for HRQoL and those for symptoms, so no conclusions can be drawn. The results on selective outcome reporting were also inconclusive.

### Comparison with previous research

In our analysis, low reporting rates of PROs in SmPCs were evident in oncologic indications, even though regulatory authorities specifically recommend considering PROs [2], for instance, to inform choices between therapies with similar efficacy results, especially in palliative settings [16]. Although comparability with previous research is limited due to methodological differences, our findings of poor reporting for PROs are generally supported, both for oncologic indications and across indications. A review of PRO labelling for oncologics approved by the US Food and Drug Administration (FDA) and the European Medicines Agency (EMA), 2012–2016, found that while no FDA PRO labelling was identified, the EMA SmPCs included PRO information for 21 (46.7%) of 45 indications. The authors explained this discrepancy by “different evidentiary standards to assess PRO data from oncology studies, with the EMA more likely to accept data from open-label studies and broad concepts such as health-related quality of life” [17].

In a review of FDA PRO labelling for 182 new drug applications across indications (2011–2015), 16.5% had PRO labelling, almost all for concepts proximal to the disease [15]. In a further review across indications, which investigated the role and extent of PRO usage within EMA European Public Assessment Reports (2008–2012), which contain an SmPC section, PROs as any outcomes were used for 82 of 180 drugs (46%) [18].

Non- or delayed reporting of PROs is also evident in journal publications. Out of 231 primary publications of oncology randomized phase III trials with HRQoL data as a secondary or exploratory outcome, HRQoL results were available in only 143 (61.9%). For trials without HRQoL results in the primary publication, probability of secondary publication with such results was 12.5% after 1 and 40.3% after 3 years [16].

With regard to selective outcome reporting, the systematic review above [16] found that the non-reporting of HRQoL data in primary publications was similar in trials with negative and positive results, indicating that the main problem seems to be underreporting, not selective reporting.

Our analysis expands on previous research by adding the HTA perspective and highlighting the value of PROs as clinically relevant outcomes for health policy decision-making.

### The role of SmPCs and dossier assessments

SmPCs can support patient education (e.g. regarding rare adverse effects) and clinical decision-making, as they often contain unique and detailed information on a drug [19]. However, their user friendliness has been criticized; for instance, advice may be vague or information difficult to find [19, 20]. User testing of the effectiveness of SmPCs in communicating essential information to prescribers found that they are of low perceived value and not central to prescribing behaviour [21]. Merely including PROs as a default in the SmPC would thus not suffice; to increase usage of PROs (and of SmPCs in general), user friendliness of SmPCs would also need to be improved. In addition, regular updating should be ensured for PRO data. The lower PRO reporting rates in SmPCs compared with dossier assessments might be explained by the traditional focus of regulators on objective clinical outcomes and laboratory parameters. Thus, even though pharmaceutical companies record PROs in clinical trials, they are not necessarily reported in SmPCs, as they are not regarded to be of major relevance for the regulatory decision-making procedure [18, 22]. We would like to note that, as an HTA agency, we have a different perspective than a drug approval authority and the question arises whether it is appropriate to apply HTA standards to a regulatory outcome document such as the SmPC. However, according to the European Commission’s SmPC guideline, regarding SmPC content “It may be appropriate to provide limited information, relevant to the prescriber, such as the main results … regarding pre-specified end points or clinical outcomes in the major trials…” [9]; in our opinion, information relevant to the prescriber must include information on PROs.

Dossier assessments, which are also available in English, may represent a valuable additional PRO source. Examples where PROs informed health policy or clinical decision-making, but were not reported in SmPCs, indicating that they did not contribute much to regulatory decision-making, are presented in Additional file 4. Overall, both the amount of PRO data considered in dossier assessments and their relevance for decisions on added benefit have increased over the years since the introduction of AMNOG [23, 24], indicating that both the generation and the quality of PRO data in clinical trials have improved. However, there is still much room for improvement here and moreover, the mere availability of high-quality PRO data does not necessarily translate into better consideration in healthcare decision-making in practice. A systematic review of the impact of PROs from clinical trials showed relatively limited evidence demonstrating real world PRO-related research impact [25]. Therefore, PROs should not only be reported more frequently, but should generally be given greater importance in treatment decisions.

### Limitations

Our sample size was relatively small and we did not conduct statistical tests, so we cannot draw any conclusions on the statistical significance of differences between our results. In addition, we only considered basic outcome categories for PROs.

## Conclusion

Even though a large amount of PRO data on new drugs is available from RCTs included in SmPCs, the corresponding results are underreported. PROs should be reported as default in SmPCs to support informed decision-making in healthcare that ensures adequate consideration of the patient perspective.

## Supplementary Information

**Additional file 1: Table.** Information evaluated in our analysis and coding options.

**Additional file 2: Figure.**Completeness of reporting of PROs in SmPCs grouped by direction of treatment effects (positive effects / no difference / negative effects)

**Additional file 3: Figure.**Completeness of reporting of PROs in SmPCs: (a) grouped by outcome category, (b) grouped by outcome category and direction of treatment effects (positive effects / negative or no effects)

**Additional file 4: Table.**Examples of dossier assessments with relevant PROs missing in the SmPC

## Data Availability

The datasets used and/or analysed during the current study are available from the corresponding author on reasonable request.

## Abbreviations

AMNOG: Arzneimittelmarktneuordnungsgesetz (Act on the Reform of the Market for Medicinal Products)
EMA: European Medicines Agency
FDA: Food and Drug Administration
G-BA: Gemeinsamer Bundesausschuss (Federal Joint Committee)
HRQoL: Health-related quality of life
IQWiG: Institut für Qualität und Wirtschaftlichkeit im Gesundheitswesen (Institute for Quality and Efficiency in Health Care)
PRO: Patient-reported outcome
RCTs: Randomized controlled trial
SmPC: Summary of product characteristics
