# Supplementary material for: Results on patient-reported outcomes are underreported in summaries of product characteristics for new drugs
Source: J Patient Rep Outcomes. 2021 Dec 7;5:127. doi: 10.1186/s41687-021-00402-1 (PMC8651888; doi:10.1186/s41687-021-00402-1)
Supplement: Supplementary file 1 — Additional file 1: Table. Information evaluated in our analysis and coding options. [file 41687_2021_402_MOESM1_ESM.docx]

Additional File 1

Table: Information evaluated in our analysis and coding options

| **Information evaluated^a^** | **Coding options** |
| --- | --- |
| **Did the RCT contain evaluable PRO data?** |  |
| PRO (HRQoL) reported in the dossier assessment | yes^b^ / no^c^ |
| PRO (symptom) reported in the dossier assessment | yes^b^ / no^c^ |
| **Was the RCT included in the dossier assessment also included in the SmPC?** | yes / no |
| **Were the PRO data from the RCT reported in the SmPC?** |  |
| PRO (HRQoL) reported in the SmPC | yes^b^ / no^c^ |
| PRO (symptom) reported in the SmPC | yes^b^ / no^c^ |
| **To what extent were the PROs reported in the SmPC^d^?** |  |
| PRO (HRQoL) | completely^e^/ partly^f^ / not reported^g^ |
| PRO (symptom) | completely^e^/ partly^f^ / not reported^g^ |
| **To what extent were the PROs reported in the SmPC based on the direction of the treatment effect^h^?** |  |
| PRO (HRQoL) |  |
| positive effect | completely^i^/ partly^j^ / not reported^k^ |
| negative or no effect^l^ | completely^i^/ partly^j^ / not reported^k^ |
| PRO (symptom) |  |
| positive effect | completely^i^/ partly^j^ / not reported^k^ |
| negative or no effect | completely^i^/ partly^j^ / not reported^k^ |
| a. Evaluation was based on the RCTs included in the dossier assessment. b. (Evaluable) PROs investigated in the included RCTs. “Evaluable” PROs means sufficient PRO data recorded with valid and suitable tools. “Sufficient” data generally means that the proportion of study participants included in the PRO analysis was at least 70% of the original study population and that the difference in the proportion of study participants excluded from the PRO analysis was smaller than 15 percentage points between the intervention and control group [1]. c. No PROs were investigated in the RCT included or PROs were investigated, but not evaluable (e.g. due to methodological problems such as missing data). d. This step only considered those RCTs in the dossier assessments investigating the target population specified in the SmPC, as in these cases, the PROs reported in the SmPC were expected to be consistent with the PROs reported in the dossier assessment.  e. All respective PROs considered in the dossier assessment were reported completely (i.e. with results) in the SmPC. f. Information on PROs reported in the dossier assessment was missing in the SmPC (e.g. only rudimentary information provided [i.e. no results or no information on subscales]).  g. None of the respective PROs reported in the dossier assessment were reported in the SmPC.  h. Positive / negative effects were defined as a result with a significance level of p < 0.05. PRO results with “no effect” were defined as results with a significance level of p ≥ 0.05.  i. All respective PROs in the effect category considered in the dossier assessment were reported completely (i.e. with results) in the SmPC. j. Information on PROs in the effect category considered in the dossier assessment was missing in the SmPC (e.g. only rudimentary information provided [i.e. no results or no information on subscales]).  k. None of the respective PROs in the effect category considered in the dossier assessment were reported in the SmPC.  l. Originally two separate categories, now pooled into one, because of only a small number of PROs with negative effects. | |
| HRQoL: health-related quality of life; PRO: patient-reported outcome; RCT: randomized controlled trial; SmPC: summary of product characteristics | |

Reference

1. Institute for Quality and Efficiency in Health Care. General Methods; Version 6.0 [online]. 2020 [Accessed: 2021 September 18]. URL: https://www.iqwig.de/methoden/general-methods_version-6-0.pdf.
