# Supplementary material for: Results on patient-reported outcomes are underreported in summaries of product characteristics for new drugs
Source: J Patient Rep Outcomes. 2021 Dec 7;5:127. doi: 10.1186/s41687-021-00402-1 (PMC8651888; doi:10.1186/s41687-021-00402-1)
Supplement: Supplementary file 2 — Additional file 2: Figure.Completeness of reporting of PROs in SmPCs grouped by direction of treatment effects (positive effects / no difference / negative effects) [file 41687_2021_402_MOESM2_ESM.docx]

Additional File 2


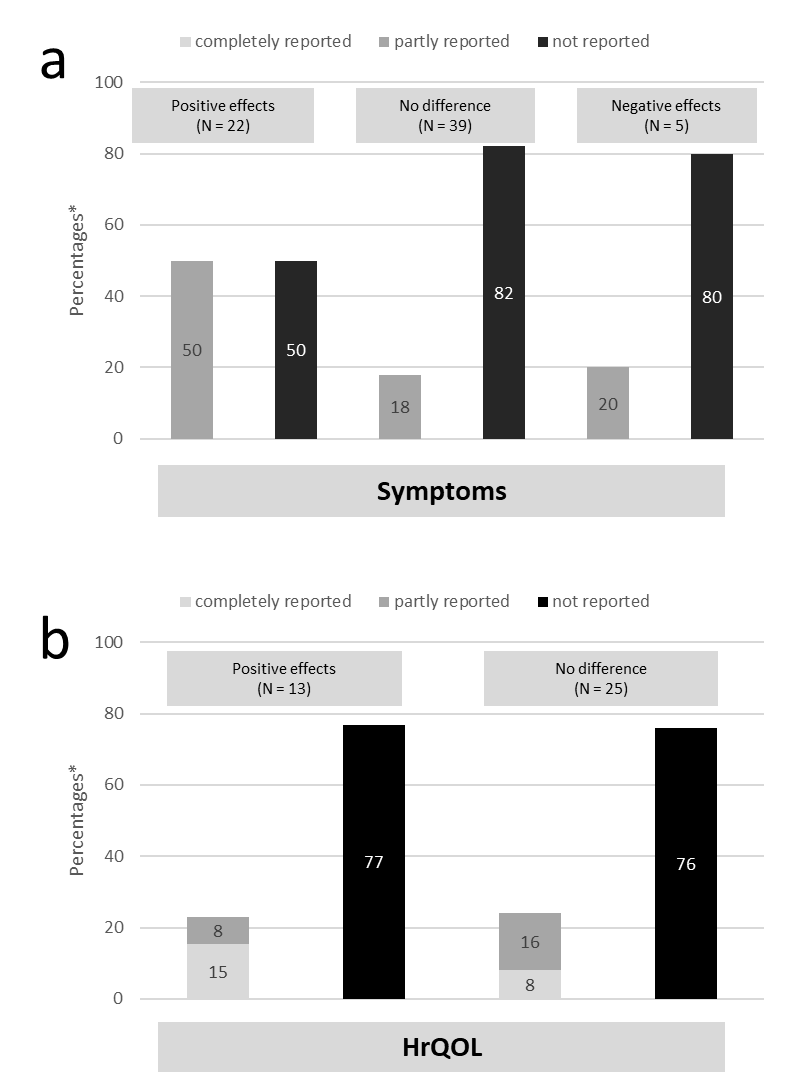


Figure: Completeness of reporting of PROs in SmPCs grouped by direction of treatment effects: (a) symptoms (b) HrQoL. Negative effects were only shown for symptoms, not for HRQoL

* Proportion of RCTs with evaluable PRO data reported in the SmPCs.

HRQoL: health-related quality of life; PRO: patient-reported outcome; RCT: randomized controlled trial; SmPC: summary of product characteristics
