# Supplementary material for: Results on patient-reported outcomes are underreported in summaries of product characteristics for new drugs
Source: J Patient Rep Outcomes. 2021 Dec 7;5:127. doi: 10.1186/s41687-021-00402-1 (PMC8651888; doi:10.1186/s41687-021-00402-1)
Supplement: Supplementary file 4 — Additional file 4: Table.Examples of dossier assessments with relevant PROs missing in the SmPC [file 41687_2021_402_MOESM4_ESM.docx]

Examples of dossier assessments with relevant PROs missing in the SmPC

| Drug | Indication | RCT(s) relevant for assessment | PROs considered in the assessment (outcome category, direction of effect) | Consequences for decision-making process |
| --- | --- | --- | --- | --- |
| Sacubitril/ Valsartan [[1-4](#_ENREF_1)] | Symptomatic chronic heart failure with reduced ejection fraction | PARADIGM-HF [[5](#_ENREF_5)] | - Health status EQ‑5D VAS (morbidity, no effect) - KCCQ OSS (HrQoL, positive effect) | The positive effects seen in the KCCQ OSS were crucial for the decision “considerable added benefit” of sacubitril/valsartan versus the ACT ACE inhibitor (enalapril). |
| Palbociclib [[6-8](#_ENREF_6)] | Postmenopausal women with HR- positive, HER2-negative advanced/ metastatic breast cancer (first-line treatment) | PALOMA-2 [[9](#_ENREF_9)] | - Health status EQ‑5D VAS (morbidity, no effect) - FACT-B (HrQoL, no effects) | There were no effects on PROs, despite a noticeable difference in disease progression. No difference in overall survival was shown, but greater harm regarding severe and serious adverse events. Overall, the absence of positive effects on PROs led to the decision “no added benefit” |
| Alectinib [[10-12](#_ENREF_10)] | First-line treatment of adult patients with ALK-positive advanced NSCLC | ALEX [[13](#_ENREF_13)] | - Health status EQ‑5D VAS (morbidity, no effect) - EORTC QLQ-LC13 and EORTC QLQ-C30 (morbidity, several positive effects [diarrhoea, nausea and vomiting, appetite loss, dysphagia], 1 negative effect [dyspnoea]) - EORTC QLQ-C30 (HrQoL, no effects) | Several positive effects and a negative effect were measured by the PRO instruments. These effects did not change the overall decision on added benefit, but were regarded to be important information for decision-making by clinicians and patients. |
| ACT: appropriate comparator therapy; ALK: anaplastic lymphoma kinase; EORTC: European Organisation for Research and Treatment of Cancer; FACT‑B: Functional Assessment of Cancer Therapy-Breast Cancer; HER2: human epidermal growth factor receptor 2; HR: hormone receptor; HRQoL: health-related quality of life; KCCQ: Kansas City Cardiomyopathy Questionnaire; NSCLC: non-small cell lung cancer; OSS: overall summary score; PRO: patient-reported outcome; QLQ-C30: Quality of Life Questionnaire-Core 30; QLQ-LC13: Quality of Life Questionnaire-Lung Cancer 13; RCT: randomized controlled trial; SmPC: summary of product characteristics; VAS: visual analogue scale | | | | |

References

1. Institut für Qualität und Wirtschaftlichkeit im Gesundheitswesen. Sacubitril/Valsartan: Nutzenbewertung gemäß § 35a SGB V; Dossierbewertung [online]. 2016 [Zugriff: 08 Apr 2016]. URL: <https://www.iqwig.de/download/A15-60_Sacubitril-Valsartan_Nutzenbewertung-35a-SGB-V.pdf>.

2. Gemeinsamer Bundesausschuss. Tragende Gründe zum Beschluss des Gemeinsamen Bundesausschusses über eine Änderung der Arzneimittel-Richtlinie (AM-RL): Anlage XII - Beschlüsse über die Nutzenbewertung von Arzneimitteln mit neuen Wirkstoffen nach § 35a SGB V – Sacubitril/Valsartan [online]. 2016 [Zugriff: 27 Sep 2021]. URL: <https://www.g-ba.de/downloads/40-268-3823/2016-06-16_AM-RL-XII_Sacubutril_Valsartan_D-207_TrG.pdf>.

3. Institute for Quality and Efficiency in Health Care. Sacubitril/valsartan – Benefit assessment according to §35a Social Code Book V [online]. 2016 [Zugriff: 27 Sep 2021]. URL: <https://www.iqwig.de/download/a15-60_sacubitril-valsartan_extract-of-dossier-assessment.pdf>.

4. Institute for Quality and Efficiency in Health Care. Sacubitril/valsartan – Addendum to Commission A15-60 [online]. 2016 [Zugriff: 07.10.2021]. URL: <https://www.g-ba.de/downloads/92-975-1411/Addendum%20zur%20Nutzenbewertung%20des%20IQWiG_Sacubitril_Valsartan.pdf>.

5. Novartis Pharmaceuticals. This Study Will Evaluate the Efficacy and Safety of LCZ696 Compared to Enalapril on Morbidity and Mortality of Patients With Chronic Heart Failure (PARADIGM-HF) [online]. [Zugriff: 27 Sep 2021]. URL: <https://clinicaltrials.gov/ct2/show/study/NCT01035255>.

6. Gemeinsamer Bundesausschuss. Tragende Gründe zum Beschluss des Gemeinsamen Bundesausschusses über eine Änderung der Arzneimittel-Richtlinie (AM-RL): Anlage XII - Beschlüsse über die Nutzenbewertung von Arzneimitteln mit neuen Wirkstoffen nach § 35a SGB V – Palbociclib [online]. 2017 [Zugriff: 27 Sep 2021]. URL: <https://www.g-ba.de/downloads/40-268-4388/2017-05-18_AM-RL-XII_Palbociclib_D-264_TrG.pdf>.

7. Institut für Qualität und Wirtschaftlichkeit im Gesundheitswesen. Palbociclib (Mammakarzinom): Nutzenbewertung gemäß § 35a SGB V; Dossierbewertung [online]. 2017 [Zugriff: 19.04.2017]. URL: <https://www.iqwig.de/download/A16-74_Palbociclib_Nutzenbewertung-35a-SGB-V_V1-0.pdf>.

8. Institute for Quality and Efficiency in Health Care. Palbociclib (breast cancer) – Benefit assessment according to §35a Social Code Book V [online]. 2017 [Zugriff: 27 Sep 2021]. URL: <https://www.iqwig.de/download/a16-74_palbociclib_extract-of-dossier-assessment_v1-0.pdf>.

9. Pfizer. A Study of Palbociclib (PD-0332991) + Letrozole vs. Letrozole For 1st Line Treatment Of Postmenopausal Women With ER+/HER2- Advanced Breast Cancer (PALOMA-2) [online]. [Zugriff: 27 Sep 2021]. URL: <https://clinicaltrials.gov/ct2/show/NCT01740427>.

10. Institut für Qualität und Wirtschaftlichkeit im Gesundheitswesen. Alectinib (nicht kleinzelliges Lungenkarzinom): Nutzenbewertung gemäß § 35a SGB V (neues Anwendungsgebiet); Dossierbewertung [online]. 2018 [Zugriff: 25 Apr 2018]. URL: <https://www.iqwig.de/download/A17-67_Alectinib_Nutzenbewertung-35a-SGB-V_V1-0.pdf>.

11. Gemeinsamer Bundesausschuss. Tragende Gründe zum Beschluss des Gemeinsamen Bundesausschusses über eine Änderung der Arzneimittel-Richtlinie (AM-RL): Anlage XII – Beschlüsse über die Nutzenbewertung von Arzneimitteln mit neuen Wirkstoffen nach § 35a SGB V – Alectinib (neues Anwendungsgebiet: Erstlinienbehandlung nicht-kleinzelliges Lungenkarzinom) [online]. 2018 [Zugriff: 27 Sep 2021]. URL: <https://www.g-ba.de/downloads/40-268-5066/2018-06-21_AM-RL-XII_Alectinib_D-326_TrG.pdf>.

12. Institute for Quality and Efficiency in Health Care. Alectinib (non-small cell lung cancer) – Benefit assessment according to §35a Social Code Book V (new therapeutic indication) [online]. 2018 [Zugriff: 27 Sep 2021]. URL: <https://www.iqwig.de/download/a17-67_alectinib_extract-of-dossier-assessment_v1-0.pdf>.

13. Hoffmann-La Roche. A Study Comparing Alectinib With Crizotinib in Treatment-Naive Anaplastic Lymphoma Kinase-Positive Advanced Non-Small Cell Lung Cancer Participants (ALEX) [online]. [Zugriff: 27 Sep 2021]. URL: <https://clinicaltrials.gov/ct2/show/NCT02075840>.
